# Can polygenic risk scores contribute to cost-effective cancer screening? A systematic review

**DOI:** 10.1016/j.gim.2022.04.020

**Published:** 2022-05-16

**Authors:** Padraig Dixon, Edna Keeney, Jenny C. Taylor, Sarah Wordsworth, Richard M. Martin

**Affiliations:** 1 Nuffield Department of Primary Care Health Sciences, University of Oxford, Oxford, United Kingdom; 2 MRC Integrative Epidemiology Unit, University of Bristol, Bristol, United Kingdom; 3 Population Health Sciences, Bristol Medical School, University of Bristol, Bristol, United Kingdom; 4 Wellcome Centre for Human Genetics, University of Oxford, Oxford, United Kingdom; 5 National Institute for Health and Care Research Biomedical Research Centre, Oxford, United Kingdom; 6 The Health Economics Research Centre (HERC), Nuffield Department of Population Health, University of Oxford, Oxford, United Kingdom; 7 National Institute for Health Research (NIHR) Health Research Protection Unit in Healthcare Associated Infections and Antimicrobial Resistance, Oxford, United Kingdom

**Keywords:** Cancer, Cost-effectiveness, Polygenic risk scores, Screening, Systematic review

## Abstract

**Purpose:**

Polygenic risk influences susceptibility to cancer. We assessed whether polygenic risk scores could be used in conjunction with other predictors of future disease status in costeffective risk-stratified screening for cancer.

**Methods:**

We undertook a systematic review of papers that evaluated the cost-effectiveness of screening interventions informed by polygenic risk scores compared with more conventional screening modalities. We included papers reporting cost-effectiveness outcomes with no restriction on type of cancer or form of polygenic risk modeled. We evaluated studies using the Quality of Health Economic Studies checklist.

**Results:**

A total of 10 studies were included in the review, which investigated 3 cancers: prostate (n = 5), colorectal (n = 3), and breast (n = 2). Of the 10 papers, 9 scored highly (score >75 on a 0-100 scale) when assessed using the quality checklist. Of the 10 studies, 8 concluded that polygenic risk-informed cancer screening was likely to be more cost-effective than alternatives.

**Conclusion:**

Despite the positive conclusions of the included studies, it is unclear if polygenic risk stratification will contribute to cost-effective cancer screening given the absence of robust evidence on the costs of polygenic risk stratification, the effects of differential ancestry, potential downstream economic sequalae, and how large volumes of polygenic risk data would be collected and used.

## Introduction

### Rationale

This paper systematically reviews the literature that assessed the use of polygenic risk data to influence the costeffectiveness of cancer screening. Genetic testing for inherited cancer susceptibility is an established part of care for individuals in whom family histories or ancestry indicate significant liability to cancer in many health care systems.^1^ For example, genetic data can be used to inform the future risk of disease as part of cancer screening programs in populations that are influenced by particular founder effects, such as screening interventions directed at identifying BRCA1/BRCA2 breast and ovarian cancer susceptibility variants among Ashkenazi Jews.^2^

However, it is increasingly appreciated that polygenic as well as monogenic risk may affect cancer incidence and its progression.^3^ Polygenic risk reflets the cumulative influence of many different sources of genetic variation on disease risk, rather than the influence only of rare pathogenic variants in single genes, eg, the BRCA1/BRCA2 genes for breast and ovarian cancer.^4^

Polygenic risk scores (PRSs) reflect the aggregated effect of many genetic variants (typically single-nucleotide variations [SNVs], or single-nucleotide polymorphisms [SNPs]) that are known to influence the incidence of cancer or some other cognate outcome.^5^ PRSs may be predictive of disease risk, even if the individual variants of which it is comprised themselves have only modest effects on risk when considered alone.^6–8^

Callender et al^9^ reported that individuals in the first and 99th percentiles for PRS of incident prostate cancer have relative risks of 0.09 and 5.52 respectively, compared with population means. In some cases, differences in relative risk are associated with potentially significant differences in absolute risk. For example, Mavaddat et al^10^ found that the estimated lifetime absolute risk of ER-positive breast cancer by age 80 years ranged between 2% for women in the lowest centile of polygenic risk and 31% for those in the highest centile.

Polygenic risk appears, in some cases, to influence risks associated with monogenic risk variants.^11,12^ Kapoor et al^13^ found that preventive strategies intended to modify individual risk factor for breast cancer could have a greater influence on absolute risk of breast cancer for women at higher polygenic risk. Among other uses of these data, they may have utility in screening programs to identify asymptomatic individuals at increased risk of disease incidence.^14–17^

Conversely, there are several reasons why polygenic risk data might not necessarily improve the effectiveness and cost-effectiveness of screening programs. Most individuals will not be in the tails of a single disease-specific polygenic risk distribution. Large interpercentile relative risks may be associated with only modest differences in absolute risk.^17,18^ These issues are compounded by the modest heritability of many cancers, and circumstances in which polygenic risk offers little incremental enhancement to risk prediction among the individuals with penetrant monogenic variants, a positive family history, or other risk factors.^19^

Screening requires clear evidence of a net benefit to the wider community to justify invasive and potentially harmful investigations, such as biopsy and the possibility of overtreatment of indolent cancers.^20^ These and other considerations reflect the Wilson-Jungner criteria^21^ for screening, which recommend that screening programs be cost-effective and are therefore efficient uses of scarce health care resources that may have more valuable alternative uses.^22^ Similarly, the European Guide on Quality Improvement in Comprehensive Cancer Control^23^ recommends that the costeffectiveness of screening interventions be evaluated before intervention implementation.

Obtaining and using polygenic risk data (especially on a large scale) may be expensive, may lead to higher rates of false positives, overtreatment, increased patient anxiety, and higher caseloads for medical professionals without clinical benefit. Moreover, population-level screening using PRSs is likely to be more complex in admixed societies because the majority of existing PRSs are more predictive of disease in individuals of European ancestry (Vassy J, Hao L, Kraft P, et al. Clinical validation, implementation, and reporting of polygenic risk scores for common diseases. unpublished preprint).

A recent review^24^ of the use of PRSs in screening found only very limited investigation of the cost-effectiveness of using PRSs in screening, and no systematic reviews of the use of PRSs in cancer screening have been undertaken. The 2021 Polygenic Risk Score Task Force of the International Common Disease Alliance^5^ noted the very limited economic evidence concerning uses of PRSs in general and recommended that research should address this as a priority.

This paper reports our systematic literature review, which assessed whether using polygenic risk data is likely to influence the cost-effectiveness of cancer screening.

### Objectives

Our objectives were to assess the extent of the literature that evaluated the use of PRSs in cost-effectiveness analyses of cancer screening, to examine how PRSs were used in costeffectiveness cancer screening models, and to evaluate how PRS data influence the cost-effectiveness of cancer screening interventions compared with non–cancer screening modalities. Screening is a process to identify individuals, who may be asymptomatic, at increased risk of disease.

We examined studies with participants undergoing population-scale screening for any form of cancer. Interventions in the scope of our review were cancer screening using PRSs compared with cancer screening without PRSs. We examined outcomes relating to cost-effectiveness, with no restrictions placed on whether cost-effectiveness analyses were informed by or conducted alongside any particular study design.

## Materials and Methods

We used the Preferred Reporting Items for Systematic Reviews and Meta-Analyses (PRISMA) 2020 systematic review guidelines^25^ to inform the development, conduct, and reporting of this study. Appendix 1 contains a completed PRISMA checklist.

### Protocol and registration

The study protocol is available on the International Prospective Register of Systematic Reviews database (https://www.crd.york.ac.uk/prospero/display_record.php?RecordID=243659) to which it was prospectively uploaded on April 1, 2021, before the first searches commenced on April 16, 2021.

### Eligibility criteria

We included studies that used PRS data in cost-effectiveness analyses of cancer screening. We considered English-language journal articles or preprints published in any country or time period. We included studies modeling any form of cancer, using PRSs in screening, and reporting costeffectiveness outcomes such as net monetary benefit or incremental cost-effectiveness ratios. Appendix 2 summarizes inclusion and exclusion criteria in more detail.

### Information sources

A systematic review of the literature using the NHS Economic Evaluation Database, Medline, Embase, Health Technology Assessment databases, National Institute for Health and Care Excellence guidelines, UK National Screening Committee guidance, preprint servers bioR and medRxiv, and hand searches were carried out, with no restriction on date. Relevant data were extracted and the results were narratively synthesized.

### Search strategies

The full search strategies for all databases are presented in Appendix 3.

### Study selection

One author (P.D.) independently selected reports that appeared to fulfill inclusion criteria on the basis of review of abstracts and titles. Papers were retained for full-text review when they appeared to meet inclusion criteria or in which there was insufficient evidence to exclude them. Two reviewers (P.D. and E.K.) then reviewed the full text of articles. After discussion and reconciliation of any discrepancies in judgments reached by the 2 reviewers, the full text of articles meeting these criteria was then subject to further review by both reviewers against all inclusion criteria.

### Data collection process

Data extraction was informed by the recommendations and example data extraction form proposed by the Centre for Reviews and Dissemination at the University of York.^26^ We piloted a spreadsheet data extraction form on a specimen cost-effectiveness analysis. We then refined this on the basis of discussions between 2 authors (P.D. and E.K.). Data were independently extracted to populate this form by P.D. and E.K.

### Data items

We obtained the following information from papers meeting all inclusion criteria.

- Study objective
- Cancer(s) studied
- Context (eg, country studied)
- Type of economic evaluation used
- Proposed design for a screening program
- Risk thresholds (if used)
- Adherence to screening
- Screening interval
- Economic model structure
- Age range of cohort
- Size of cohort modeled
- Perspective of the analysis
- Cancer treatments
- Modeling of cancer progression
- Mortality measures
- Health state utility values
- Duration of follow up
- Outcome measure
- How genetic data (assumed to be) were obtained and analyzed
- Assumptions made in creating the polygenic risk score
- PRS and related costs
- How PRS data were included and modeled
- Treatment of ancestral background, ethnicity, and race
- Cost-effectiveness threshold
- Cost-effectiveness results of PRS-informed screening compared with non-PRS screening modalities
- Sensitivity of cost-effectiveness results

### Risk of bias and quality assessment in individual studies

We appraised the quality of included studies using the Quality of Health Economics Studies (QHES) checklist.^27^ This is not a tool for assessing risk of bias per se, but was found to have the highest construct validity among 79 tools for assessing the quality of cost-effectiveness studies in a recent systematic review.^28^

The QHES uses a weighted grading system to measure the quality of studies against 16 criteria, each of which is weighted by importance. The full list of criteria and their weights is presented in Appendix 4. The range of the QHES score is from 0 to 100; values >75 indicate a study of high quality. Each study was assessed using the QHES checklist by 1 author (P.D.).

### Summary measures

We extracted data on the cost-effectiveness of cancer screening interventions using PRSs in comparison to interventions without PRSs. This included data on incremental cost-effectiveness ratios, net benefit, and related summary measures of intervention cost-effectiveness. We also extracted and summarized information on the methods through which PRS data were used in each cost-effectiveness model implementation as detailed earlier in data items section.

### Synthesis of results and additional analyses

A narrative synthesis of results was undertaken. No additional analysis such as subgroup analysis or meta-regression was planned or undertaken. Where necessary, we referred to literature cited in included studies to clarify any missing or unclear data issues. Certainty assessment involved consideration of the limitations of the included literature.

## Results

### Study selection

**Figure 1:**
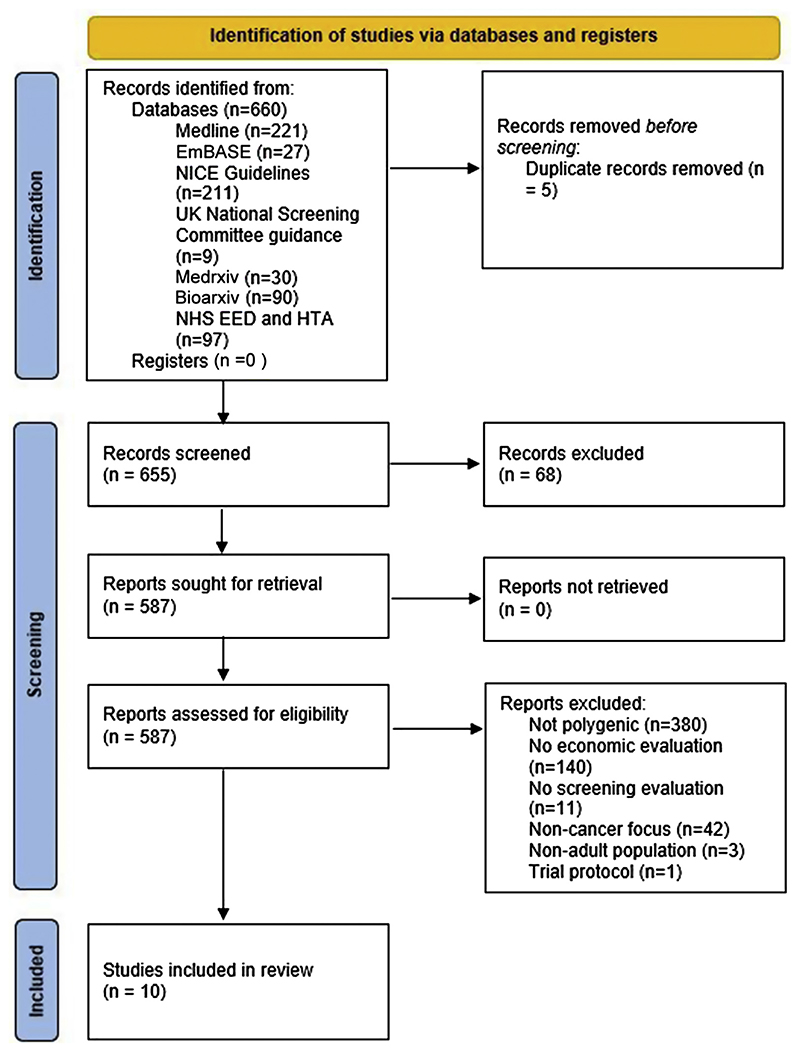
Identification of studies. EED, Economic Evaluation Database; HTA, Health Technology Assessment; NICE, National Institute for Health and Care Excellence.

Searches concluded in June 2021 and reflect literature that met the inclusion criteria up to that point. The systematic search identified 660 articles (Figure 1, based on the PRISMA 2020 statement^25^).

Excluding duplicates, the titles and abstracts of 655 articles were screened, from which 68 records were excluded because they were denoted as review articles (n = 63), or comprised only an abstract (n = 5). After this step, 587 records were successfully sought for retrieval and assessed for eligibility. A total of 577 records were then excluded, the most frequent reasons for which were an absence of polygenic focus or the absence of an economic evaluation. In total, 10 studies that met inclusion criteria were included in the review.

### Study characteristics

Table 1 summarizes some of the key characteristics of each of the 10 included papers. A full table detailing these and many further study characteristics is presented in Appendix 5.

Included papers were recently published, with the oldest paper published in 2018. Three cancers were studied in these 10 papers: prostate cancer (n = 5), colorectal cancer (n = 3), and breast cancer (n = 2). All studies used a costutility design, in which costs were related to quality-adjusted life years (QALYs). This permitted the comparison of the cost-effectiveness of these cancer screening programs with other types of intervention in different clinical areas. A total of 7 studies used a health system perspective to define the scope of costs to be included in these analyses. The exceptions were Naber et al^36^ and Hao et al^29^ (both health system and societal perspectives) and Karlsson et al^30^ (societal perspective).

These cost-utility models were implemented using cohort models (n = 6) or microsimulation models (n = 4). Both cohort models and microsimulation models involve a dynamic simulation of health and disease processes over time. Microsimulation models generally permit greater flexibility in the modeling of event timing and with respect to the interdependency of events.^38^ In both types of models, each simulated individual sojourns for a period of time in different states of health, to which are attached state-specific costs and quality of life values. These state-specific values are combined with the amount of person-time spent in each state to produce cohort-level cost-effectiveness parameters, which enabled comparisons of different screening strategies.

Conventional cost-effectiveness thresholds (which are used to inform funding decisions in health technology assessments) were applied in the 7 papers that used these thresholds in ex-ante analysis. Wong et al^33^ and Naber et al^36^ calculated cost-effectiveness thresholds in relation to other model parameters. Hendrix et al^31^ compared strategies on the basis of incremental cost-effectiveness ratios and the associated concept of dominance.

Baseline health state utility values were drawn in 6 papers from population values. The exceptions were Wong et al,^33^ Hendrix et al,^31^ Naber et al,^36^ and Cenin et al.^35^ Adjustments were made in all papers for utility in different health states, and for disease progression, interventions, and treatments. No adjustments, whether positive or negative, were made to utility as a result of an individual’s knowledge of their own polygenic risk in any of the papers.

In each type of model, cancer treatments were typically defined by the stage of cancer and by applicable local or national guidelines on cancer care. The microsimulation models generally offered more detailed modeling of the natural history of cancer progression. The populations that were modeled reflected profiles of those typically eligible for inclusion in cancer screening in the specific jurisdiction studied, and followed up simulated individuals for an appropriate amount of time, including lifetime follow ups. The screening intervals that were modeled broadly reflected actual or plausible “real world” screening implementations.

None of the papers described in any detail how genetic data for the entire modeled cohort would be acquired. There may be significant effects on cost-effectiveness depending on when and where in the clinical pathway the sample to create the PRS would be obtained, which genotypes would be assessed, how the results would be interpreted, and how findings would be communicated. Some papers (eg, Hao et al^29^ Karlsson et al^30^) noted a primary care consultation as a first step in the process.

There was no modeling of the effect of differential polygenic risk by ancestry. In total, 6 papers modeled the effect of lower take-up or imperfect adherence to screening; the exceptions were Wong et al,^33^ Hao et al,^29^ Karlsson et al,^30^ and Hendrix et al.^31^

Various means of including polygenic risk and assessing their influence in relation to risk thresholds or other indicators for intervention were implemented. Wong et al^33^ created tertiles of a hypothetical polygenic risk distribution to identify low, medium, and high polygenic risk groups. Hendrix et al^31^ evaluated the effect of the proprietary Prompt Prostate Genetic Score. The number of alleles was not described, but references in that paper suggest that it was based on 29 prostate cancer SNVs in 4528 men of European ancestry in the placebo arm of the Prostate Cancer Prevention Trial.^39^

Hao et al^29^ and Karlsson et al^30^ evaluated the Stock-holm3 package. This model combines measurement of prostate-specific antigen (PSA), protein biomarkers, PRS (based on 232 SNVs), and clinical information collected using questionnaire, including age, family history, and previous prostate biopsies.^40^ These variables have all been reported to be associated with prostate cancers with a Gleason score of >7.0, and the Stockholm3 model has been shown to outperform PSA testing alone in predicting prostate cancers with Gleason score of >7.0.^40^ Karlsson et al^30^ evaluated screening strategies that used Stockholm3 test as a reflex test (ie, tests prompted after particular levels of PSA) for PSA values ≥ 1, 1.5, and 2 ng/mL. Hao et al^29^ implemented a similar model with a screening strategy that used Stockholm3 with reflex test thresholds of PSA values ≥1.5 and 2 ng/mL.

Naber et al^36^ generated a relative risk distribution with different values of the area under the curve for a polygenic test ranging from 0.60 to 0.80 in each simulated, hypothetical cohort. These cohorts were split in 60 groups defined by their relative risk of colorectal cancer.

Thomas et al^32^ modeled random assignment of risk alleles to individuals to reflect allele frequencies obtained from UK Biobank data, as well as correlations between alleles on the same chromosome. Genetic^41^ and nongenetic risk factors were combined to obtain individualized relative risks for colorectal cancer, which were applied to transition probabilities from normal epithelium to adenoma and to colorectal cancer. Estimated relative risk was adjusted to ensure that the simulated distribution of disease reflected expected colorectal cancer incidence.

Cenin et al^35^ stratified their cohort population into 5 risk groups on the basis of quintiles of polygenic risk and fist degree family history of colorectal cancer. The prevalence of the 5 categories was simulated given a probability of being in any SNV quintile and of positive first-degree family history. The relative risk of colorectal cancer, compared with average population risk, was based on combined relative risk of each polygenic risk quintile and family history.

Pashayan et al,^37^ Callender et al,^9^ and Callender et al^34^ followed a similar approach. Given a log-additive model of interaction between genetic and conventional nongenetic risk factors, the distribution of risk for disease incidence was log-normal on a relative risk scale. Callender et al^9^ used data from Schumacher et al^42^ and Dadaev et al.^43^ Percentile ranks associated with relative risk or absolute risk were obtained given knowledge of the mean and variance of the log-normal relative risk distribution.

### Risk of bias/study quality within studies

We evaluated all studies using the QHES checklist.^27^ Appendix 6 details the score awarded to each study against the 16 criteria of this checklist. The QHES checklist does not identify the presence or scale of biases that may affect the conclusions of the included studies. However, it does permit a characterization of the quality of the economic evaluation and the implications that this may have for the robustness of the results.

All but one study (Wong et al^33^) received a score indicating high study quality (>75 on a 0-100 scale). The study by Wong et al^33^ did not reach the threshold for a number of reasons, but included the absence of (1) remission from cancer and (2) the possibility of between-state transitions from their model. We concluded that even the highest scoring studies did not account for all potential downstream effects of PRS-related interventions. This is an inherently difficult task but its identification does indicate the qualifications that must be used in interpreting each paper.

### Results of individual studies and synthesis of results

We extracted summary information on the costeffectiveness of polygenic risk-informed screening strategies compared with strategies that did not use these data. It was not feasible to explore heterogeneity in the costeffectiveness conclusions across studies given the relatively small number of papers meeting inclusion criteria and given the narrow range of cancers studied. Direct comparison across studies was also complicated by the differences in how cost-effectiveness results were reported, by the differences in the ways in which polygenic risk was incorporated into wider risk models, and by the types of strategy compared.

Of the 10 studies, 8 concluded that polygenic risk-informed screening was likely to be cost-effective, or had the lowest incremental cost-effectiveness ratio of strategies evaluated. Naber et al^36^ and Cenin et al^35^ did not conclude that polygenic risk-informed screening was likely to be cost-effective.

Conclusions in all papers were conditional on important model parameters including the cancer studied, comparator interventions, age at which screening commences, and screening intervals. Where assessed, sensitivity analyses of specific model parameters were typically deterministic or one-way. This approach can be misleading given correlations between parameters and the nonlinear structure of the various simulation models used.^44^

## Discussion

### Summary of evidence

We conducted and reported a systematic review of the cost-effectiveness of using polygenic risk data in population-scale cancer screening in comparison to more conventional screening modalities. The evidence base in this area is both recent and relatively small. We identified 10 studies encompassing 3 different cancers that met our inclusion criteria. Most studies concluded that the use of polygenic risk to inform risk stratification for cancer screening was likely to be cost-effective.

Each study used varieties of simulation models (either cohort Markov models or microsimulation models) to capture the dynamic process of disease over time in costutility frameworks. Of the 10 studies, 9 were judged to be of high quality when assessed against the QHES checklist, although these and similar checklists do not capture all considerations relevant to the implementation of PRSs in cancer screening.

### Limitations

#### Limitations of the evidence

The most robust source of evidence on the long-term effect on cost-effectiveness of using polygenic risk data in cancer screening would come from trials with very long follow up of mortality and other outcomes. In the absence of data from such trials, cost-utility simulation models of the type meeting the inclusion criteria of this review are likely to constitute the best available alternative means of evaluating how PRSs might contribute to cost-effective cancer screening.

##### Downstream consequences

There is a need for greater evidence on the economic consequences of using PRS in screening, including their acquisition costs, costs incurred alongside genetic data collection and risk stratification, downstream economic sequelae, and remuneration for their integration into routine care. Included studies did not examine in depth any service redesigns that may be necessary to implement this type of screening. These could include changes in rates of consultation under more widespread use of polygenic risk data and changes in prescribing behavior influenced by pharmaco-genetic considerations.

##### Costs of PRS

All studies lacked robust data on the per-individual costs of polygenic risk stratification for use in large-scale screening programs. Studies either assumed a cost for obtaining the information necessary to undertake polygenic risk-informed screening, or back calculated the costs at which such screening might alter estimated cost-effectiveness. Most papers (other than Hendrix et al^31^ and Naber et al^36^) parameterized uncertainty around the mean costs of polygenic risk stratification or used a threshold analysis (as in Thomas et al^32^ and Cenin et al^35^) to examine the sensitivity of cost-effectiveness results to its cost.

##### Acquiring and using genetic data on a large scale

None of the papers described in any detail how genetic data for the entire modeled cohort would be acquired. There was some mention of samples being taken by buccal swab (eg, Wong et al^33^) or that any test to collect these data would be administered by a general practitioner (eg, Cenin et al^35^) but there was little to no other consideration given to how these data would be collected at scale, and the economic implications of so doing.

##### Ancestry

There was little to no attention given to the differential availability and predictive capacity of PRSs by ancestry. For example, Callender et al^9^ studied prostate cancer screening for middle-aged men in England, and assumed all such men have access to the same PRS. This implicitly assumes the availability of a European-ancestry PRS, whereas approximately 7% to 8% of men in the target age groups studied in that paper did not report this ancestry in the 2011 UK census.^45^

##### Cancer incidence and cancer aggression

The principal focus of all included studies was on SNVs that predict disease incidence. This is associated with 2 potential limitations. The first is that such studies may exhibit a survival bias by preferentially including in their analysis individuals who avoided lethal disease. The extent of this bias is unknown, and will depend to some degree on the natural history of each cancer. The second potential limitation relates to the value of identifying SNVs that predict disease incidence as opposed to those that identify disease progression or disease aggression. The extent of overlap and positive correlation between germline liability to disease incidence and these other phenotypes would mean that the relative balance of lethal and indolent cancers that are identified by a PRS-informed screening program could potentially be altered. For some individuals with penetrant monogenic variants, it appears that PRSs also further influence overall absolute disease risk.^12^ However, the extent to which such a balance between over- and undertreatment is achieved, and indeed whether the use of PRSs may ultimately worsen overdiagnosis and overtreatment, will depend on the specific ways in which a screening program is designed and implemented.

##### Structural and parameter uncertainty

There was little to no assessment of the sensitivity of costeffectiveness for different ways of modeling PRSs. Parameter uncertainty was accounted to some degree by probabilistic sensitivity analysis,^22,46^ but uncertainty about model structure was (at best) approached by investigating results under different assumptions and without necessarily characterizing the plausibility of the scenarios modeled under these assumptions.

### Limitations of the systematic review

This review assessed literature published or uploaded to preprint servers in English language by June 2021. The quality assessment/risk of bias assessment was undertaken only by a single author. Only 3 cancers were studied in the 10 papers included in the review, and it was not possible to infer whether cancer type influenced the probability of polygenic risk-informed screening being cost-effective.

The identification of new variants influencing disease and the development of new methods to estimate PRSs may also alter the balance of evidence in this area, particularly if new scores are more predictive. However, most variants with large effect sizes on disease incidence have probably been uncovered (at least in well-studied populations of European ancestry) and further progress is likely to come from identification of germline variants associated with aggressive cancers.

As the literature develops and the number of papers meeting inclusion criteria grows, it may be feasible to examine potential modifiers of screening cost-effectiveness. These may include the type of cancer studied, the broad structure of the screening interventions modeled, the health system in which the screening is to be performed, and the age structure of the population modeled.

We did not have information on the scale and scope of any publication bias in this area. We did not plan to conduct meta-analysis given anticipated heterogeneity in cancer type, model structure, and the basis on which polygenic risk was modeled. Instead, we have provided a high-level summary of the cost-effectiveness of polygenic risk-informed modalities compared with more conventional screening strategies, but it was not feasible to summarize all possible combinations of screening strategies.

Finally, although we used the QHES checklist, which was assessed as having the highest sensitivity of any such checklist included in the systematic review and assessment of Walker et al,^28^ it does not and cannot identify all issues that may affect the quality and robustness of any particular study.

### Implications for future research

There is scope to expand the number of cancers studied in this literature, and to extend and improve the sources used to populate the parameters of these models to address parameter uncertainty. Future models should also consider how more detailed modeling of natural histories, new screening modalities, and diagnostic pathways may influence structural uncertainty although this will likely require some contextual nuance because increasing the complexity of a model does not necessarily secure its robustness or relevance.

Recent methodological developments (eg, McCabe et al^47^) support the greater use of probabilistic sensitivity analysis in cost-effectiveness analysis. There is also scope for the development of standards that could guide the reporting of economic evaluations of PRS-informed risk-stratified screening.

Identification of SNVs that predict aggressive disease could have a material effect on the cost-effectiveness of large-scale screening programs, particularly if SNVs associated with disease aggression are distributed across centiles of a PRS that predicts incidence.^48,49^ The use of PRSs that predict disease incidence, progression and aggression may have multiple effects on the relative balance of overdiagnosis and overtreatment but this will ultimately depend on the specific ways in which a screening program is designed and implemented.

It is also important to note that different methods for calculating PRSs at the level of individual, which will be necessary for risk stratification, may not be stable across methods (in the sense that an individual’s percentile rank may change according to the approach used) and will be associated with uncertainty. Ding et al^50^ found large variance in individual PRS estimates, and recommended a probabilistic approach to polygenic risk stratification that estimates the probability that an individual’s PRS is more than a prespecified risk threshold for screening.

There is a need to establish how the cost-effectiveness of screening might vary by ancestral background given that most available PRSs are most predictive for individuals of European ancestry (Vassy J, Hao L, Kraft P, et al. Clinical validation, implementation, and reporting of polygenic risk scores for common diseases. 2021). Future research may also consider the equity effect of introducing new tests that disproportionately benefits relatively more privileged groups. This could be facilitated by the use of comparative modeling as undertaken by, eg, the National Institutes of Health Cancer Intervention and Surveillance Network.^51^ Future modeling could also consider the effect of possible intervention-generated inequalities,^52^ and the effect of knowledge of one’s own PRS on anxiety, participation in screening, and other outcomes.^53,54^

A further broad area for future research relates to the costing and valuing of polygenic risk data. All studies necessarily lacked robust external data on the per-individual costs of population-level genotyping and on all downstream economic impacts stemming from the use of these data. Further research is therefore required on the cost of obtaining comprehensive PRS for deployment at population scale.

## Conclusion

The use of polygenic risk data in population-level screening for cancer is attracting increasing interest. A major concern with using these data in population-level screening will be their cost-effectiveness. The literature on this topic is recent, relatively small, and examines only 3 cancers, albeit that these cancers are relatively prevalent and have screening programs in some countries. Of the 10 included studies, 8 concluded that the use of polygenic risk data would likely be cost-effective. However, these conclusions should be evaluated in research that addresses specific limitations and expands the scope of this literature. This is likely to require prospective evidence from randomized controlled trials, as well as comprehensive economic decision analytic models where several data sources, including randomized controlled trials, are synthesized and modeled.

## Supplementary Material

Supplementary material

## Figures and Tables

**Table 1:** Selected characteristics and outcomes of included studies

Study	Year Published	Cancer	Country	Type of Model	N of Cohort Modeled	Age Profiles Modeled	Cost of PRS	Cost-Effectiveness Results
Hao et al^29^	2022	Prostate	Sweden	Microsimulation model (Prostata model)	10 million	From age 55 and followed up through remainder of lifetime	€251 including prostate-specific antigen (PSA) test analysis, GP visit, and test analysis	Stockholm3 test with a reflex threshold of PSA value ≥2 ng/ mL had the lowest ICER, €38,894 per QALY gained, in the base case analysis. Note: reflex testing refers to further diagnostic testing that may be prompted by an elevated PSA level.
Karlsson et al^30^	2021	Prostate	Sweden	Microsimulation model (Prostata model)	Not directly stated but references related work that refers to cohorts of 100 million men	From birth and followed up over lifetime	€255 (including GP visit)	Prostate cancer screening using the polygenic risk-informed Stockholm3 test for men with an initial PSA value of ≥2.0 ng/mL was cost-effective compared with screening using only PSA.
Hendrix et al^31^	2021	Prostate	United States	Microsimulation model (Fred Hutchinson Cancer Research Centre model)	100 million	Age 40 years (with different screening start ages of >40 years) and followed up till age 100 years. Screening assumed to stop at age 69 years	$250 based on commercial costs of the Prompt-PGS software	Cost-effectiveness of PRS- informed risk screening compared with universal screening depended on universal screening policy modeled. PRS-informed risk- stratified screening most likely to be cost-effective when universal screening is performed on an annual basis starting at age 55 years.
Thomas et al^32^	2021	Colorectal	England	Microsimulation model (MiMiCBowel).	6,787,000	Age ≥30 years, screening taking place at various ages depending on the strategy, risk-assessment assumed to be carried out at age 40 years.	No costs assigned to risk scoring, instead, cost analysis was carried out to determine maximum justifiable cost of implementing risk scoring in population at age 40 years.	PRS-informed screening was very likely to be cost-effective when used in conjunction with phenotypic information compared with screening strategies relying on phenotypic data alone.
Wong et al^33^	2021	Breast	Singapore	Markov model	3,014,388 individuals included in the model, Not otherwise reported	Women aged between 35 and 74 years	Genotyping of buccal swab assumed to cost SGD 210.	Compared with biennial mammogram-only screening, polygenic-risk informed screening had lower costs and higher QALYs and was very likely to be cost-effective.
Callender et al^34^	2021	Prostate	England	Life table cohort Markov model	4.48 million	Screening took place at age 55 to 69 years with follow up till age 90 years	£25 based on personal communication of tariffs used in the English National Health Service	Multiparametric MRI-first risk- stratified screening scenarios at risk thresholds of >3.5% were more cost-effective than no screening at a costeffectiveness threshold of £20,000. Strategies with highest net monetary benefit at cost-effectiveness thresholds of £20,000 and £30,000 were MRI-first risk- stratified screening at risk thresholds of 8.5% and 7.5%, respectively.
Cenin et al^35^	2020	Colorectal	Australia	Microsimulation model (the MISCAN-Colon model)	100 million	Age 40 years (and born in 1980) and followed up until age 100 years, at which point, individuals in the cohort were assumed to be dead, screening assumed to stop at age 74 years	Assumed cost £200 based on a commercially available polygenic test for breast cancer	Uniform screening was more likely to be cost-effective than PRS-informed risk-based screening. Personalized and uniform screening scenarios yielded similar QALYs. Personalized screening cost more than uniform screening, largely owing to the cost of determining risk.
Naber et al^36^	2019	Colorectal	United States	Microsimulation model (the MISCAN-Colon model)	Cohort described as consisting of >1 million simulated individuals, not otherwise reported	Age 40 years with US life expectancy, and followed up until death, screening modeled as ending between age 70 and 85 years	Assumed cost £200 based on currently available commercial polygenic tests	Polygenic risk-informed screening was unlikely to be cost-effective; this form of screening yielded same number of QALYs as uniform screening at increased costs.
Callender et al^9^	2019	Prostate	England	Life table cohort Markov model	4.48 million	Screening took place at age 55 to 69 years with follow up till age 90 years	£25, estimated from personal discussion of costs charged to NHS hospitals for prostate cancer genome wide associations studies	Risk-based screening was costeffective at a costeffectiveness threshold of £20,000 per QALY gained compared with no screening at all 10-year absolute risk thresholds of >4.5%. At all 10-year absolute risk of <10%, risk-based screening led to a greater number of incremental QALYs gained than age-based screening while incurring fewer additional costs at all risk thresholds >2%.
Pashayan _et al_^37^	2018	Breast	England	Life table cohort model	364,500	Age 50 years with follow up till age 85 years	£50, based on per variant research cost of genotyping	PRS-informed risk stratification at the 70th risk percentile had the highest net monetary benefit, with a 72% probability of being costeffective at a costeffectiveness threshold of £20,000.

ICER, incremental cost-effectiveness ratio; GP, general physician or general practitioner; MiMiC, microsimulation model in cancer; MISCAN, microsimulation screening analysis; MRI, magnetic resonance imaging; PRS, polygenic risk scores; QALY, quality-adjusted life years; SGD, Singapore dollar.

## Data Availability

The search terms used to interrogate each database are presented in Appendix 3. No other code was used in this review. Template data collection forms are available from the corresponding author on request. All data extracted from each included study are available in the supplementary material.
