# Supplementary material for: Can polygenic risk scores contribute to cost-effective cancer screening? A systematic review
Source: Genet Med. Author manuscript; Available in PMC 2023 Feb 24. (PMC7614235; doi:10.1016/j.gim.2022.04.020)
Supplement: Supplementary material [file EMS170524-supplement-Supplementary_material.pdf]

Appendix 1 – Completed PRISMA 2020 systematic review checklist

Section and Topic	Item #	Checklist item	Location where item is reported
**TITLE**			
Title	1	Identify the report as a systematic review.	Page 1
**ABSTRACT**			
Abstract	2	See the PRISMA 2020 for Abstracts checklist.	Page 2
**INTRODUCTION**			
Rationale	3	Describe the rationale for the review in the context of existing knowledge.	Page 3-5
Objectives	4	Provide an explicit statement of the objective(s) or question(s) the review addresses.	Page 5
**METHODS**			
Eligibility criteria	5	Specify the inclusion and exclusion criteria for the review and how studies were grouped for the syntheses.	Page 6-7
Information sources	6	Specify all databases, registers, websites, organisations, reference lists and other sources searched or consulted to identify studies. Specify the date when each source was last searched or consulted.	Page 7-8
Search strategy	7	Present the full search strategies for all databases, registers and websites, including any filters and limits used.	Appendix 2
Selection process	8	Specify the methods used to decide whether a study met the inclusion criteria of the review, including how many reviewers screened each record and each report retrieved, whether they worked independently, and if applicable, details of automation tools used in the process.	Page 8
Data collection process	9	Specify the methods used to collect data from reports, including how many reviewers collected data from each report, whether they worked independently, any processes for obtaining or confirming data from study investigators, and if applicable, details of automation tools used in the process.	Page 9
Data items	10a	List and define all outcomes for which data were sought. Specify whether all results that were compatible with each outcome domain in each study were sought (e.g. for all measures, time points, analyses), and if not, the methods used to decide which results to collect.	Page 8-9
	10b	List and define all other variables for which data were sought (e.g. participant and intervention characteristics, funding sources). Describe any assumptions made about any missing or unclear information.	Page 8-9
Study risk of bias assessment	11	Specify the methods used to assess risk of bias in the included studies, including details of the tool(s) used, how many reviewers assessed each study and whether they worked independently, and if applicable, details of automation tools used in the process.	Page 9-10
Effect measures	12	Specify for each outcome the effect measure(s) (e.g. risk ratio, mean difference) used in the synthesis or presentation of results.	Page 10

UOB Open

Section and Topic	Item #	Checklist item	Location where item is reported
Synthesis methods	13a	Describe the processes used to decide which studies were eligible for each synthesis (e.g. tabulating the study intervention characteristics and comparing against the planned groups for each synthesis (item #5)).	Page 10
	13b	Describe any methods required to prepare the data for presentation or synthesis, such as handling of missing summary statistics, or data conversions.	Page 10
	13c	Describe any methods used to tabulate or visually display results of individual studies and syntheses.	Page 10
	13d	Describe any methods used to synthesize results and provide a rationale for the choice(s). If meta-analysis was performed, describe the model(s), method(s) to identify the presence and extent of statistical heterogeneity, and software package(s) used.	Page 10
	13e	Describe any methods used to explore possible causes of heterogeneity among study results (e.g. subgroup analysis, meta-regression).	Page 10
	13f	Describe any sensitivity analyses conducted to assess robustness of the synthesized results.	Page 10
Reporting bias assessment	14	Describe any methods used to assess risk of bias due to missing results in a synthesis (arising from reporting biases).	N/A
Certainty assessment	15	Describe any methods used to assess certainty (or confidence) in the body of evidence for an outcome.	Page 10
**RESULTS**			
Study selection	16a	Describe the results of the search and selection process, from the number of records identified in the search to the number of studies included in the review, ideally using a flow diagram.	Page 10-12
	16b	Cite studies that might appear to meet the inclusion criteria, but which were excluded, and explain why they were excluded.	N/A
Study characteristics	17	Cite each included study and present its characteristics.	Page 12-13
Risk of bias in studies	18	Present assessments of risk of bias for each included study.	Page 18-19 and Appendix 4
Results of individual studies	19	For all outcomes, present, for each study: (a) summary statistics for each group (where appropriate) and (b) an effect estimate and its precision (e.g. confidence/credible interval), ideally using structured tables or plots.	Appendix 3
Results of syntheses	20a	For each synthesis, briefly summarise the characteristics and risk of bias among contributing studies.	Page 17-18
	20b	Present results of all statistical syntheses conducted. If meta-analysis was done, present for each the summary estimate and its precision (e.g. confidence/credible interval) and measures of statistical heterogeneity. If comparing groups, describe the direction of the effect.	N/A
	20c	Present results of all investigations of possible causes of heterogeneity among study results.	N/A
	20d	Present results of all sensitivity analyses conducted to assess the robustness of the synthesized results.	N/A
Reporting biases	21	Present assessments of risk of bias due to missing results (arising from reporting biases) for each synthesis assessed.	N/A

UOB Open

Section and Topic	Item #	Checklist item	Location where item is reported
Certainty of evidence	22	Present assessments of certainty (or confidence) in the body of evidence for each outcome assessed.	N/A
**DISCUSSION**			
Discussion	23a	Provide a general interpretation of the results in the context of other evidence.	Page 18-23
	23b	Discuss any limitations of the evidence included in the review.	Page 18-23
	23c	Discuss any limitations of the review processes used.	Page 20-21
	23d	Discuss implications of the results for practice, policy, and future research.	Page 21-23
**OTHER INFORMATION**			
Registration and protocol	24a	Provide registration information for the review, including register name and registration number, or state that the review was not registered.	Page 2
	24b	Indicate where the review protocol can be accessed, or state that a protocol was not prepared.	Page 2
	24c	Describe and explain any amendments to information provided at registration or in the protocol.	N/A
Support	25	Describe sources of financial or non-financial support for the review, and the role of the funders or sponsors in the review.	Page 2
Competing interests	26	Declare any competing interests of review authors.	Page 24
Availability of data, code and other materials	27	Report which of the following are publicly available and where they can be found: template data collection forms; data extracted from included studies; data used for all analyses; analytic code; any other materials used in the review.	Page 24

UOB Open

Appendix 2 – Inclusion and exclusion criteria

Table A1 **Summary of inclusion and exclusion criteria**

Inclusion criteria (papers included if they meet all criteria)	Exclusion criteria (papers excluded if any of these criteria are encountered)
**Participants**: Studies including participants undergoing screening for any form of cancer	**Participants**: Participants screened for diseases other than cancer; non-human studies
**Intervention & Comparison**: Use of PRS in cancer screening cost-effectiveness evaluation compared to any other non-PRS screening modality	**Intervention & Comparison**: Use of monogenic rather than polygenic tests; no use of genetic tests Interventions not involving screening
**Outcomes**: Cost-effectiveness outcome statistics and other outcomes detailed in Section 2.2	**Outcomes**: Studies not reporting cost-effectiveness outcomes
**Study design**: Cost-effectiveness analysis reporting an outcome such as an incremental cost-effectiveness ratio, net monetary benefit, net health benefit. Trial or model- based evaluation using data from any study design	**Study design**: Studies other than cost-effectiveness analyses; cost-effectiveness work not reporting comparisons of incremental measures of cost and effect.
**Other criteria:** Any country, health system, or time period; Journal articles; preprints, English language	**Other criteria**: Grey literature. Books. Not in English language. Expert opinion, abstracts, conference proceedings, methodological, general, commentary and review articles not containing original research.

UOB Open

Appendix 3 – Search strategies

Medline

----

1 cost-effectiveness.mp. or Cost-Benefit Analysis/

2 (cost adj3 (effect$ or util$)).tw.

3 cancer.mp. or *Neoplasms/

4 screening.mp. or *Mass Screening/

5 (Screen$ or test$).tw.

6 Polymorphism, Single Nucleotide/ or Genetic Predisposition to Disease/ or Multifactorial Inheritance/ or Genome-Wide Association Study/

7 1 or 2

8 4 or 5

9 3 and 6 and 7 and 8

Embase

----

1 cost-effectiveness.mp. or exp "cost effectiveness analysis"/

2 (cost adj3 (effect$ or util$)).tw.

3 cancer.mp. or *malignant neoplasm/

4 screening.mp. or cancer screening/ or DNA screening/ or genetic screening/ or screening/ or mass screening/ or screening test/

5 (Screen$ or test$).tw.

6 genetic risk score/ or polygenic.mp. or multifactorial inheritance/ (16396)

7 1 or 2

8 4 or 5

9 3 and 6 and 7 and 8

CRD HTA,DARE and NHS EED

UOB Open

Results for: ((cancer) AND (screening) AND (polygenic OR genetic)) and ((Economic evaluation:ZDT and Bibliographic:ZPS) OR (Economic evaluation:ZDT and Abstract:ZPS) OR Project record:ZDT OR Full publication record:ZDT)

Medrxiv

"cancer AND cost-effectiveness AND polygenic"

Biorxiv

"cancer AND cost-effectiveness AND polygenic"

National Institute for Health and Care Excellence (NICE)

Searched all published NICE guidelines for the word "cancer"

UK National Screening Committee

Searched for recommendations pertaining to cancer in adult populations

UOB Open

Appendix 4 – Specimen Quality of Health Economic Studies (QHES) checklist

	Questions	Points available	Yes	No
1.	Was the study objective presented in a clear, specific, and measurable manner?	7		
2.	Were the perspective of the analysis (societal, third-party payer, etc.) and reasons for its selection stated?	4		
3.	Were variable estimates used in the analysis from the best available source (RCT = best, expert opinion = worst)?	8		
4.	If estimates came from a subgroup analysis, were the groups pre-specified at the beginning of the study?	1		
5.	Was uncertainty handled by (1) statistical analysis to address random events, (2) sensitivity analysis to cover a range of assumptions?	9		
6.	Was incremental analysis performed between alternatives for resources and costs?	6		
7.	Was the methodology for data extraction (including the value of health states and other benefits) stated?	5		
8.	Did the analytic horizon allow time for all relevant and important outcomes? Were benefits and costs that went beyond 1 year discounted (3% - 5%) and justification given for the discount rate?	7		
9.	Was the measurement of costs appropriate and the methodology for the estimation of quantities and unit costs clearly described?	8		
10.	Was the primary outcomes measure(s) for the economic evaluation clearly stated and were the major short-term, long-term and negative outcomes included?	6		
11.	Were the health outcomes measures/scales valid and reliable? If previously tested valid and reliable measures were not available, was justification given for the measures/scales used?	7		
12.	Were the economic model (including structure), study methods and analysis, and the components of the numerator and denominator displayed in a clear, transparent manner?	8		
13.	Were the choice of economic model, main assumptions, and limitations of the study stated and justified?	7		
14.	Did the author(s) explicitly discuss the direction and magnitude of potential biases?	6		

UOB Open

15.	Were the conclusions/recommendations of the study justified and based on the study results?	8		
16.	Was there a statement disclosing the source of funding for the study?	3		
Total score		100		

UOB Open

Appendix 5 – Detailed outcomes for all papers

	Hao et al, 2021	Karlsson et al, 2021	Hendrix et al, 2021	Thomas et al, 2021	Wong et al, 2021	Callender et al, 2021	Cenin et al, 2020	Naber et al, 2019	Callender, 2019	Pashayan et al, 2018
Study objective	To assess the cost-effectiveness of quadrennial magnetic resonance imaging (MRI)-based screening using either Stockholm3 (S3M) or prostate-specific antigen (PSA) test as a reflex test.	To assess the cost-effectiveness of the Stockholm3 Model (S3M) in screening.	To assess the cost-effectiveness of moving from universal screening (risk-agnostic) to risk-stratified screening	To use risk scores as the basis for determining age at which faecal immunochemical test (FIT) screening should start, then to estimate the cost-effectiveness, clinical benefits and resource impact of polygenic risk informed - stratification, compared with current screening strategies.	To evaluate the cost-effectiveness of a breast cancer screening programme that incorporates genetic testing against the current biennial mammogram-only screening programme.	To evaluate the benefit, harm and cost-effectiveness of MRI before biopsy compared with biopsy-first screening for prostate cancer using age-based and polygenic risk-stratified screening strategies.	To investigate the impact of personalizing colorectal cancer screening, based on polygenic risk and family history and to compare its cost-effectiveness to uniform screening (using fecal immunochemical testing (FIT) and colonscopies at different intervals and different starting ages)	To assess whether and under what conditions polygenic risk-informed screening for colorectal cancer may be a cost-effective alternative to uniform screening (which involved colonoscopy screening at ages 50,60 and 70 years).	To assess the benefit-harm ratio and cost-effectiveness of a polygenic risk-tailored screening programme for prostate cancer	To assess the benefit/harm ratio and cost-effectiveness of polygenic risk-stratified breast screening compared with standard age based screening and no screening.
Cancer(s) studied	Prostate	Prostate	Prostate	Colorectal	Breast	Prostate	Colorectal	Colorectal	Prostate	Breast
Context (screening strategies compared and country)	Swedish setting. Strategies compared: No screening MRI for PSA≥3ng/mL and TBx/SBx for PI-RADS 3-5 MRI for S3M≥15% using a reflex threshold of PSA≥1.5ng/mL and TBx/SBx for	Sweden setting. Strategies compared: No prostate cancer screening; screening using the PSA test; and screening using the S3M test as a reflex test for PSA values ≥ 1, 1.5 and 2 ng/mL	US setting Strategies compared: No screening and nine combinations of starting age (45, 50 or 55) and screening interval (1, 2 or 4 years). Strategies compared for each risk stratum separately. Then	England setting. Biennial faecal immunochemical test (FIT), starting at an age determined through polygenic-informed risk-assessment at age 40, compared to FIT screening that started at a fixed	Singapore setting. Strategies compared: biennial mammogram with polygenic-risk informed screening.	England setting. Strategies compared: No screening Age-based screening with biopsy if PSA ≥ 3, age-based screening with MRI if PSA ≥ 3 and biopsy if abnormal findings	Australia setting Strategies compared: No screening, plus 25 different screening strategies defined by different start ages for screening (40, 46, 50, 54, or 60 years), test used (FIT or	US setting Strategies compared: No screening, risk-stratified screening based on PRS, Uniform screening with colonoscopies at ages 50, 60, and 70 years.	England setting Strategies compared: no screening, age-based screening with prostate-specific antigen (PSA) testing PRS-informed risk-tailored screening	UK setting. Strategies compared: No screening, Age-based screening, PRS-informed risk stratified screening

	men who had PI-RADS 3-5<br>MRI for S3M≥15% using a reflex threshold of PSA≥2ng/mL and TBx/SBx for men who had PI-RADS 3-5		compared universal policies to risk-stratified policies in which intermediate-risk men are screened with the same intensity as in the universal policy and low- and high-risk men receive lower and higher intensity screening, respectively.	age for all individuals.<br><br>Baseline strategy based on current FIT screening strategy in England involving biennial FIT at a threshold of 120µg/g between ages of 60 and 74. Three other comparators involved a start age of 50 instead of 60, the use of threshold of 20µg/g.		Risk-stratified screening with PRS as well as age, biopsy if PSA ≥ 3<br>Risk-stratified screening with PRS, MRI if PSA ≥ 3 and biopsy if abnormal findings	colonoscopy), and interval (annual, biennial or<br>triennial screening for FIT, and every 5 or 10 years for colonoscopy).			
Type of economic evaluation used	Cost-utility	Cost-utility	Cost-utility	Cost-utility	Cost-utility	Cost-utility	Cost-utility	Cost-utility	Cost-utility	Cost-utility
Proposed design for a polygenic risk-informed screening programme	Screening assumed to be administered by general practitioners but otherwise not described.	Not described	Not described	Not described	Assumed that buccal swabs would be collected and used in genotyping. Also asked to complete a questionnaire on breast cancer risk factors, before being stratified into three risk groups based on their initial PRS. Individuals in	Not described.	Not described	Assumes that population (at average risk for colorectal cancer) that is willing to undergo polygenic test as well as subsequent risk-informed colonoscopy screening.	Not described.	Not described.

					each risk group will receive their initial PRSs within three to 6 months of a buccal swab.					
Risk thresholds (if used)	Screening based on PSA≥3ng/mL; and S3M risk with reflex test thresholds of PSA≥1.5 and 2ng/mL. S3M risk prediction based on a genetic risk score based on a single nucleotide polymorphism (SNP) chip, five plasma protein biomarkers, together with self-reported age, family history and any previous	Men with an S3M risk of Gleason 7 cancer above 10% referred to a urologist. negative biopsies	Pre-specified thresholds of the Prompt-PGS © (Prompt-Prostate Genetic Score (≤0.60, >0.6–1.3, and >1.3)) used to designate participants as belonging to low-, intermediate-, and high-risk strata.	Different risk scores were used based on combinations of phenotypic and genetic information. The age for a first FIT invite was calculated as the age at which an individual would reach a specific risk threshold (separately for each risk score) at age 40. This ensured that number of FIT screenings was similar between comparator and intervention screening strategies.	Three groups were defined for the intervention defined by percentile cutoffs of polygenic risk: PRS stratified as <60% as low risk, 60-95th percentile as high risk, and >95% for high risk. Different cutoffs for the risk groups evaluated in a scenario analysis.	Varied the 10-year absolute risk of developing prostate cancer (based on age and polygenic risk) from 2% to 10% in men aged between 55 and 69.	Using previous research, the population was stratified into five risk groups based on quintiles of polygenic risk score.	Each population was split into relative risk groups, into which individuals were assigned based on baseline risk and discriminative accuracy of the polygenic test.	Varied the 10-year absolute risk of developing prostate cancer based on PRS thresholds at which individuals were eligible for screening from 2% to 10%	Risk threshold used in polygenic risk-informed risk based screening in which only the proportion of women in the population with a risk score greater than a threshold risk were offered screening. Women were screened every three years until age 69 if they met a polygenic-risk informed threshold. 99 scenarios of risk-stratified screening strategies corresponding to percentiles of the risk score were evaluated.
Adherence to screening	Assumed entire cohort would participate in screening.	Assumed entire cohort would participate in screening.	Assumed entire cohort would participate in screening.	One screening scenario reduced modelled screening update by 25% to account for uncertainty of	Assumed entire cohort would participate in screening.	Assumed entire cohort would participate in screening in base case analysis. Sensitivity analysis varied	Base case analysis assumed perfect adherence. Subsequently estimated costs and effects of	In base-case analysis, assumed full adherence to polygenic testing, colonoscopy screening, and	Assumed entire cohort would participate in screening in base case analysis. Sensitivity analysis varied	Assumed entire cohort would participate in screening in base case analysis. In sensitivity analysis

				the impact of risk stratification, and otherwise screening uptake is assumed to be unaffected by risk stratification although update varies by age, sex, deprivation and prior response to screening.		uptake of both PSA and polygenic risk stratification to 75%.	screening at adherence levels currently observed in Australia.	colonoscopy surveillance. Sensitivity analyses modelled observed adherence rates in the US	uptake of both PSA and polygenic risk stratification to 75%.	examined impact of 90% and 75% adherence to the screening recommendation for higher and lower risk groups.
Screening intervals modelled	Quadrennial between ages of 55 and 69.	Quadrennial between ages of 55 and 69	Annual, biennial and quadrennial.	Biennial, reflecting current FIT screening strategy in England.	Biennial screening for conventional mammogram screening. Polygenic-risk informed screening may comprise self-examination, or annual, biennial or triennial screening depending on risk score.	Quadrennial between ages of 55 and 69.	Dependent on technology. Annual, biennial or triennial screening for FIT, and quinquennial or decennial for colonoscopy	Screening intervals from 1 to 20 years modelled (at intervals of 1, 2, 3, 5, 7, 10, 15, and 20 years.)	Quadrennial age-based PS from 55 to 69 under age-based screening, and quadrennial PSA testing when risk threshold reached for men aged 55 to 69 when under PRS-informed risk-tailored screening	Triennial from age 50 to 69 under both age-based screening and PRS-informed risk-based screening once risk threshold was met.
Structure of the model	Microsimulation model (Prostata model)	Microsimulation model (Prostata model)	Microsimulation model (Fred Hutchinson Cancer Research Centre model)	Microsimulation model (MiMiC-Bowel).	Markov model	Life table cohort Markov model	Microsimulation model (the MISCAN-Colon model)	Microsimulation model (the MISCAN-Colon model)	Life table cohort Markov model	Lifetable cohort model
Age range of cohort	From age 55 and followed to remainder of lifetime.	From birth and followed over lifetime.	40 years of age (with different screening start ages >40) and followed until age 100. Screening	30 and over. Screening takes place at various ages depending on strategy. Risk-assessment assumed to be	Women aged between 35 and 74	Screening took place at 55-69 years of age with follow-up to 90 years of age	40 years of age (and born in 1980) and followed until age 100, at which point individuals in the cohort	40 years of age with US life expectancy, and followed until death. Screening modelled as ending between	Screening took place at 55-69 years of age with follow-up to 90 years of age	50 years of age with follow-up to 85 years.

			assumed to stop at age 69.	carried out at age 40.			were assumed to be dead. Screening assumed to stop at age 74.	70 and 85 years of age.		
Size of cohort modelled	10 million.	Not directly stated but references related work which refers to cohorts of 100m men	100 million	6,787,000	3, 014,388 individuals included in models. Not otherwise reported	4.48 million	100 million	Polygenic risk cohort described as consisting of >1m simulated individuals. Not otherwise reported.	4.48 million	364,500
Perspective of the analysis	Both societal and healthcare perspectives	Societal perspective	Not explicitly stated but only health system costs included in analysis	Health system perspective	Health system perspective	Health system perspective	Health system perspective	Modified societal perspective comprising direct medical costs and time costs for patients and patient escorts. Non-health care costs such as travel costs were not included.	Health system perspective	Health system perspective
Cancer treatments modelled	Active surveillance, radical prostatectomy, radiation therapy	Active surveillance, radical prostatectomy and radiation therapy	Primary surgery, radiation therapy, active surveillance for low grade cancer, second-generation androgen receptor inhibitors for distant stage cancer	Treatments defined by stage of cancer. Patients found to have adenomas undergo polypectomy and applicable guidelines are implemented in the model for surveillance following adenoma removal	Treatments not specified.	Active surveillance, radical prostatectomy, radical radiotherapy, brachytherapy, chemotherapy, androgen deprivation therapy.	Treatments defined by stage and location of cancer in Australian cancer care.	Treatments defined by stage of cancer, but not otherwise specified.	Treatments based on the National Institute for Health and Care Excellence (NICE) prostate cancer pathway and NICE prostate cancer guideline. Active surveillance, radical prostatectomy, radical radiotherapy, brachytherapy, chemotherapy,	Treatment of primary breast cancer and treatment of advanced metastatic cancer, but not otherwise specified.

									androgen deprivation therapy.	
Modelling of cancer progression	Progression modelled between preclinical states (T1-T2, T3-T4 and metastasis)	Pre-clinical progression for T- and M-stage for a given Gleason score prior to diagnosis modelled.	Progression from localized to metastatic prostate cancer within Gleason grades 2-6, 7, or 8-10 modelled as well as progression from preclinical to clinical states.	Patients assumed to have normal colorectal epithelium at age 30 and then transition through nine possible states ranging from healthy epithelium to low and high risk adenoma, colorectal stages A to D , death from colorectal cancer or death from other causes. Serrated adenoma pathway modelled by transition directly from normal epithelium to CRC stage A.	Patients can transition from the healthy state to breast cancer stages I-IV or death. Patients cannot experience remission and do not transition between cancer stages.	Cancer progression not explicitly modelled	Natural history modelling of cancer progression (no lesion to screen-detectable adenoma phase (based on size of adenoma), screen detectable cancer phase (stages I to IV), to clinical colorectal stages (stages I to IV). As each simulated person ages, one or more adenomas may arise and some can progress in size from small (<5 mm) to medium (6–9 mm) to large (>10 mm).	Adenomas can progress from small (5 mm), to medium (6–9 mm), to large size (10 mm). Some adenomas can develop into cancer, which may progress through preclinical and clinical colorectal cancer stages I to IV.	Cancer progression not modelled due to uncertainty about transition rates between states and about impact of polygenic risk on these transitions	Not explicitly modelled, although model accounts for primary breast cancer and advanced metastatic breast cancer.
Mortality measures considered	Prostate cancer death and death from other causes	Prostate cancer death and death from other causes	Prostate cancer death and death from other causes.	Colorectal cancer deaths and deaths from other causes	Breast cancer death and deaths from other causes.	Prostate cancer death and death from other causes	Colorectal cancer death and death from all other causes	Colorectal cancer death and death from all other causes	Prostate cancer death and death from all other causes	Breast cancer death and death from all other causes
Health state utility values considered (for example the sources used and any anxiety associated with a	Based on general population health values Utility decrement assigned to PSA test, biopsy, cancer diagnosis,	Based on general population health values Utility decrement assigned to PSA test, biopsy, cancer diagnosis,	The “healthy state” was assigned a value of 1.0. There was no adjustment for age or for any utility impact	Age and sex-adjusted population figures were adjusted by adjustments for colorectal cancer	Stage-specific utility values were calculated from Wong et al. The “healthy state” was assigned a value	Age-adjusted utility values from the general population. Specific reduction in utility only for	Source or level of background utility values not described. Assumed utility loss due to	Source or level of background utility values not described. Assumed utility loss due to colonoscopy,	Background age-specific utility estimates, modified where necessary by a prostate treatment value	Background age-specific utility estimates, modified where necessary by a utility decrement associated with a

high-risk PRS diagnosis)	treatment/active surveillance, metastatic disease, post-recovery, palliative therapy and terminal illness. Potential psychological impacts of screening are not included	treatment/active surveillance, post-recovery, palliative therapy and terminal illness. Potential psychological impacts of screening are not included	from screening. Decrements were applied for biopsy, surveillance, treatment, symptomatic cancer and distant and end-of life states.	stages. Adjustments were made for bowel perforation and intestinal bleed. Assumed no disutility from determining or knowing polygenic risk score.	of 1.0. There was no adjustment for age or for any utility impact from determining or knowing polygenic risk score.	those with prostate cancer.	(colorectal cancer) screening colonoscopy, complication of colonoscopy and colorectal cancer care. Assumed no disutility from determining or knowing polygenic risk score.	complications of colonoscopy and colorectal cancer care by stage. Assumed no disutility of obtaining or knowing polygenic risk.	and for post-treatment recovery. Assumed no disutility of obtaining or knowing polygenic risk.	diagnosis of breast cancer. Assumed no disutility of obtaining or knowing polygenic risk.
Duration of follow-up modelled	Lifetime, from age 55.	Lifetime, from birth.	60 years from 40 to 100 years of age.	Lifetime follow-up.	40 years of follow-up until age 74	35 years of follow-up, or until age 90 years, whichever was first	60 years of follow-up until death or 100 years of age	Lifetime follow-up, starting at 40 years of age.	35 years of follow-up, or until age 90 years, whichever was first	35 years of follow up from 50 years to 85 years of age.
Outcome measure (for example cost per Quality Adjusted Life Year gained)	Costs per quality-adjusted life year.	Costs per quality-adjusted life year.	Costs per quality-adjusted life year.	Costs per quality adjusted life year.	Costs per quality adjusted life year.	Costs per quality-adjusted life year	Costs per quality-adjusted life year	Costs per quality-adjusted life year	Costs per quality-adjusted life year	Costs per quality-adjusted life year
How are genetic data obtained (or assumed to be obtained) and analyzed?	Not reported – assumed genetic data available for all men in cohort	Not reported – assumed genetic data available for all men in cohort	Not reported – assumed genetic data available for all individuals in cohort	Not reported. Risk assessment assumed to be carried out in all modelled individuals at age 40. Method of assessment not reported.	Individuals genotyped by buccal swab but not otherwise specified.	Not reported – assumed genetic data available for all men in cohort	Assumed that risk was determined by an assessment for family history and polygenic testing prior to screening. Assumed colorectal cancer family history would be taken by a general practitioner.	Not reported – assumed genetic data available for all individuals in cohort	Not reported – assumed genetic data available for all men in cohort	Not reported - assumed genetic data available for all women in cohort
Assumptions made in creating the polygenic risk score	Used the Stockholm3 (S3M) risk-model that combines	Used the Stockholm3 (S3M) risk-model that combines	The Prompt-PGS® risk score is a weighted count of Prostate	Based on 120 risk colorectal cancer risk alleles	A polygenic risk score was not used. Instead, percentiles of a	Based on 175 prostate cancer susceptibility loci identified in	Based on 45 SNPs known to increase risk of colorectal cancer	Created a hypothetical population with individual-	Number of loci not specified but based on Schumacher et	Based on 310 known breast cancer

	PSA, SNPs and other established and new plasma biomarkers. The polygenic risk score is based on 232 SNPs (Gronberg et al)	PSA, SNPs and other established and new plasma biomarkers. The polygenic risk score is based on 232 SNPs (Gronberg et al)	cancer risk-associated single nucleotide polymorphism alleles, where the weights reflect both the odds ratio of Prostate cancer diagnosis and the allele frequency in a population.	identified in Huyghe et al	an otherwise unspecified polygenic risk distribution were used to create low, medium and high risk groups. Polygenic risk was modelled as a "multiplier" that influenced higher or lower transitions from the healthy state to cancer depending on risk group membership.	genome-wide association studies. Loci assumed to interact log additively to define a lognormal distribution of polygenic risk in the population on a relative risk scale.		specific risk to which genetic variants were added until the area under the curve (AUC) of a polygenic test reached a pre-specified value. This predicted risk was then divided by population prevalence to create a relative risk, which was categorized into 60 groups.	al., and Dadaev, et al. Loci assumed to interact log additively to define a lognormal distribution of polygenic risk in the population on a relative risk scale.	susceptibility loci.
Cost of the PRS and any associated costs	€251 including PSA test analysis, GP visit and S3M test analysis	€255 (S3M test including GP visit)	$250. Based on commercial costs of the Prompt-PGS software.	No costs assigned to risk scoring. Instead, cost analysis carried out to determine maximum justifiable cost of implementing risk-scoring in population at age 40.	Genotyping of buccal swab assumed to cost SGD210.	£25. Based on personal communication of tariffs used in the English National Health Service.	Assumed cost ($200) based on a commercially available polygenic test for breast cancer	Assumed cost ($200) based on currently available commercial polygenic tests.	£25. Estimated from personal discussion of costs charged to NHS hospitals for prostate cancer genome wide associations studies.	£50. Based on per variant research cost of genotyping
How were PRS data included and modelled?	A positive S3M test was defined as one having a PSA value above the reflex threshold and a risk prediction above 10%.	A positive S3M test was defined as one having a PSA value above the reflex threshold and a risk prediction above 10%.	Estimated hazard ratios for incidence within strata of risk defined by pre-specified Prompt-PGS risk scores to identify those at low, intermediate and high risk relative to the	Each modelled individual was randomly assigned risk alleles to reflect allele frequency in UK Biobank data, and accounting for correlations between alleles	Individuals were stratified into three risk groups based on their initial PRS – low, intermediate, and high. The PRSs are stratified by setting cutoffs at below 60th	From log relative risk distribution derived the age-specific proportion of men above each 10-year absolute risk threshold, and proportion of all cancers that would be	Relative risk (compared to population average risk) of developing colorectal cancer was based on a combination of family history and quintile of PRS distribution.	A relative risk distribution was generated in hypothetical populations with varying area under the curve (AUC) values of polygenic testing of	From log relative risk of prostate cancer for each risk threshold relative to the background 10-year absolute risk of developing this cancer in the absence of screening. The	Assumed log-additive interactions between genetic and other risk factors to obtain a log-normal distribution of risk on the relative risk scale.

			average risk population.	on same chromosome.	percentile for the low-risk group, 60th to 95th percentile for the intermediate-risk group and above 95th percentile for the high-risk group. Transition probabilities between health and disease states influenced by "multipliers" of 2x, 1x ,and 0.5x for each group.	diagnosed in these men. These proportions were used to calculate the age-specific relative risk of developing prostate cancer in those men above and below the 10-year absolute risk thresholds.	Family history and quintile of PRS risk were observed to be largely independent.	0.60, 0.65, 0.70, 0.75, and 0.80. This population was split into groups of estimated relative risk, which assigned individuals to a relative risk group based on baseline risk and accuracy of the polygenic test.	log relative risk of developing prostate cancer was then applied to the polygenic risk distribution to determine the proportion of cases above the threshold. This was used to derive the relative risk of developing prostate cancer amongst the screened and unscreened.	Percentile rank of relative risk or (age-conditional) absolute risk was calculated. Calculated the relative risk associated with a risk score in higher- and lower-risk subgroups in relation to a relative risk distribution.
Whether ethnicity was considered in relation to PRS, and whether differential cost-effectiveness was considered by ethnicity	Ethnicity not considered	Ethnicity not considered	Ethnicity not considered.	Ethnicity was included as a phenotypic risk factor. Differential cost-effectiveness by ethnicity not considered.	Percentile risk group definitions were adjusted to account for Asian ancestry. Ethnicity not otherwise considered.	Ethnicity not considered.	Ethnicity not considered.	Not explicitly modelled, although some discussion of how adherence may vary by ethnicity	Ethnicity not considered.	Ethnicity not considered.
Cost-effectiveness threshold used	€47,218 (SEK 500,000) per QALY	€0 per QALY and €50,000 per QALY	A formal ex ante cost-effectiveness threshold not used – instead, strategies compared on the basis of incremental cost-effectiveness ratios	£20,000 and £30,000 per QALY both used as cost-effectiveness thresholds.	An ex-ante cost-effectiveness threshold was not used. Different thresholds were calculated to assess the probability of polygenic risk-informed	£20,000 and £30,000 per QALY	AUS$50,000 per QALY	Cost-effectiveness threshold for PRS-informed risk stratification was set equal to level at which QALYs gained were equivalent to those of uniform screening. $69,000,	£20,000 and £30,000 per QALY	£20,000 and £30,000 per QALY

					screening being cost-effective.			$65,000, $56,700, $46,000, and $38,500 for AUC of 0.60, 0.65, 0.70, 0.75, and 0.80, respectively		
Cost-effectiveness results of PRS-informed screening compared to non-PRS screening modalities	Stockholm3 with a reflex threshold of PSA≥2ng/mL had the lowest ICER, €38,894 per QALY gained, in the base case analysis	Prostate cancer screening using the polygenic risk-informed S3M test for men with an initial PSA ≥ 2.0 ng/mL was cost-effective compared with screening using only PSA.	Cost-effectiveness of PRS-informed risk screening compared to universal screening depended on universal screening policy modelled. PRS informed risk-stratified screening most likely to be cost-effective when universal screening is performed on an annual basis starting at age 55.	PRS-informed screening was very likely to be cost-effective when used in conjunction with phenotypic information compared to screening strategies relying on phenotypic data alone.	Compared with biennial mammogram-only screening, polygenic-risk informed screening had lower costs and higher quality-adjusted life years and was very likely to be cost-effective.	MRI-first risk-stratified screening scenarios at risk thresholds ≥3.5% were more cost-effective than no screening at a cost-effectiveness threshold of £20,000. Strategies with highest net monetary benefit at cost-effectiveness thresholds of £20,000 and £30,000 were MRI-first risk-stratified screening at risk thresholds of 8.5% and 7.5%, respectively	Uniform screening was more likely to be cost-effective than PRS-informed risk-based screening. Personalized and uniform screening scenarios yielded similar QALYs. Personalized screening cost more than uniform screening, largely due to the cost of determining risk.	Polygenic risk-informed unlikely to be cost-effective; this form of screening yielded same number of QALYs as uniform screening at increased costs.	Risk-based screening was cost-effective at a cost-effectiveness threshold of £20,000 per QALY gained compared to no screening at all 10-year absolute risk thresholds above 4.5%. At all 10-year absolute risk < 10%, risk-based screening led to a greater number of incremental QALYs gained than age-based screening whilst incurring fewer additional costs at all risk thresholds above 2%.	PRS-informed risk stratification at the 70th percentile had the highest net monetary benefit, with a 72% probability of being cost-effective at a at a cost-effectiveness threshold of £20,000.
Sensitivity of cost-effectiveness results to model parameters.	Reducing the unit cost of Stockholm3 to €94 (57% reduction)	The S3M test was more cost effective at higher	The cost of the polygenic risk scoring, biopsy and surveillance influence the	Risk assessment costs >£114 would not be cost effective. Cost-	Results were not sensitive to several key model parameters	MRI-first risk-stratified screening strategies were sensitive to the	Results were sensitive to the cost of determining polygenic risk. A	Risk-stratified screening could be considered cost-effective if polygenic testing	Risk-stratified screening strategies were somewhat sensitive to the	Polygenic-informed risk stratification was somewhat less likely to be cost

UOB Open

	resulted in a 16% reduction in the ICER.<br>Results sensitive to the discount rates used.	reflex thresholds, at higher biopsy costs and at lower S3M test costs	relative cost-effectiveness compared to universal age-based screening.	effectiveness was lower when risk scores were less discriminatory, with lower mean start ages for screening, or higher FIT thresholds. Men were more likely to benefit from risk-stratified screening than women.	including the low- and high-risk multipliers, direct medical costs for Stage II breast cancer,<br>and the sensitivity of mammogram and ultrasound tests.	cost of polygenic risk stratification (varied from £25 to £100). MRI-first risk-stratified screening was insensitive to a 75% (baseline 100%) uptake of polygenic risk stratification.	threshold analysis found that the costs of determining polygenic risk should not exceed $47.52 for risk-informed screening to be cost-effective at a cost-effectiveness threshold of $50,000.	costs were 30% less expensive, if the AUC of polygenic testing increased by 0.05, or a greater than 5% increase in screening adherence.	cost of polygenic risk stratification (varied from £25 to £50) and to incomplete adherence.	effective the higher the cost of the risk assessment and the lower the levels of adherence.

References

1. Grönberg, H., et al., *Prostate cancer screening in men aged 50-69 years (STHLM3): a prospective population-based diagnostic study.* Lancet Oncol, 2015. **16**(16): p. 1667-76.
2. Huyghe, J.R., et al., *Discovery of common and rare genetic risk variants for colorectal cancer.* Nature Genetics, 2019. **51**(1): p. 76-87.
3. Schumacher, F.R., et al., *Association analyses of more than 140,000 men identify 63 new prostate cancer susceptibility loci.* Nature Genetics, 2018. **50**(7): p. 928-936.
4. Dadaev, T., et al., *Fine-mapping of prostate cancer susceptibility loci in a large meta-analysis identifies candidate causal variants.* Nature Communications, 2018. **9**(1): p. 2256.

Appendix 6 – Completed QHES checklist for all included studies

Quality of Health Economic Studies (QHES) instrument – Hao et al 2021

	Questions	Points available	Yes	No
1.	Was the study objective presented in a clear, specific, and measurable manner?	7	x	
2.	Were the perspective of the analysis (societal, third-party payer, etc.) and reasons for its selection stated?	4	x	
3.	Were variable estimates used in the analysis from the best available source (RCT = best, expert opinion = worst)?	8	x	
4.	If estimates came from a subgroup analysis, were the groups pre-specified at the beginning of the study?	1	x (not applicable)	
5.	Was uncertainty handled by (1) statistical analysis to address random events, (2) sensitivity analysis to cover a range of assumptions?	9	x	
6.	Was incremental analysis performed between alternatives for resources and costs?	6	x	
7.	Was the methodology for data extraction (including the value of health states and other benefits) stated?	5	x	
8.	Did the analytic horizon allow time for all relevant and important outcomes? Were benefits and costs that went beyond 1 year discounted (3% - 5%) and justification given for the discount rate?	7	x	
9.	Was the measurement of costs appropriate and the methodology for the estimation of quantities and unit costs clearly described?	8	x	
10.	Was the primary outcomes measure(s) for the economic evaluation clearly stated and were the major short-term, long-term and negative outcomes included?	6		x
11.	Were the health outcomes measures/scales valid and reliable? If previously tested valid and reliable measures were not available, was justification given for the measures/scales used?	7	x	
12.	Were the economic model (including structure), study methods and analysis, and the components of the numerator and denominator displayed in a clear, transparent manner?	8	x	
13.	Were the choice of economic model, main assumptions, and limitations of the study stated and justified?	7	x	
14.	Did the author(s) explicitly discuss the direction and magnitude of potential biases?	6	x	
15.	Were the conclusions/recommendations of the study justified and based on the study results?	8	x	
16.	Was there a statement disclosing the source of funding for the study?	3	x	
Total score		100	94	

Quality of Health Economic Studies (QHES) instrument – Karlsson et al 2021

	Questions	Points available	Yes	No
1.	Was the study objective presented in a clear, specific, and measurable manner?	7	x	
2.	Were the perspective of the analysis (societal, third-party payer, etc.) and reasons for its selection stated?	4	x	
3.	Were variable estimates used in the analysis from the best available source (RCT = best, expert opinion = worst)?	8	x	
4.	If estimates came from a subgroup analysis, were the groups pre-specified at the beginning of the study?	1	x (not applicable)	
5.	Was uncertainty handled by (1) statistical analysis to address random events, (2) sensitivity analysis to cover a range of assumptions?	9	x	
6.	Was incremental analysis performed between alternatives for resources and costs?	6	x	
7.	Was the methodology for data extraction (including the value of health states and other benefits) stated?	5	x	
8.	Did the analytic horizon allow time for all relevant and important outcomes? Were benefits and costs that went beyond 1 year discounted (3% - 5%) and justification given for the discount rate?	7	x	
9.	Was the measurement of costs appropriate and the methodology for the estimation of quantities and unit costs clearly described?	8	x	
10.	Was the primary outcomes measure(s) for the economic evaluation clearly stated and were the major short-term, long-term and negative outcomes included?	6		x
11.	Were the health outcomes measures/scales valid and reliable? If previously tested valid and reliable measures were not available, was justification given for the measures/scales used?	7	x	
12.	Were the economic model (including structure), study methods and analysis, and the components of the numerator and denominator displayed in a clear, transparent manner?	8	x	
13.	Were the choice of economic model, main assumptions, and limitations of the study stated and justified?	7	x	
14.	Did the author(s) explicitly discuss the direction and magnitude of potential biases?	6	x	
15.	Were the conclusions/recommendations of the study justified and based on the study results?	8	x	
16.	Was there a statement disclosing the source of funding for the study?	3	x	
Total score		100	94	

Quality of Health Economic Studies (QHES) instrument – Hendrix et al 2021

	Questions	Points available	Yes	No
1.	Was the study objective presented in a clear, specific, and measurable manner?	7	x	
2.	Were the perspective of the analysis (societal, third-party payer, etc.) and reasons for its selection stated?	4	x	
3.	Were variable estimates used in the analysis from the best available source (RCT = best, expert opinion = worst)?	8	x	
4.	If estimates came from a subgroup analysis, were the groups pre-specified at the beginning of the study?	1	x (not applicable)	
5.	Was uncertainty handled by (1) statistical analysis to address random events, (2) sensitivity analysis to cover a range of assumptions?	9	x	
6.	Was incremental analysis performed between alternatives for resources and costs?	6	x	
7.	Was the methodology for data extraction (including the value of health states and other benefits) stated?	5		x (choice of perfect health for the healthy state not justified in paper)
8.	Did the analytic horizon allow time for all relevant and important outcomes? Were benefits and costs that went beyond 1 year discounted (3% - 5%) and justification given for the discount rate?	7	x	
9.	Was the measurement of costs appropriate and the methodology for the estimation of quantities and unit costs clearly described?	8	x	
10.	Was the primary outcomes measure(s) for the economic evaluation clearly stated and were the major short-term, long-term and negative outcomes included?	6		x
11.	Were the health outcomes measures/scales valid and reliable? If previously tested valid and reliable measures were not available, was justification given for the measures/scales used?	7	x	
12.	Were the economic model (including structure), study methods and analysis, and the components of the numerator and denominator displayed in a clear, transparent manner?	8	x	
13.	Were the choice of economic model, main assumptions, and limitations of the study stated and justified?	7	x	
14.	Did the author(s) explicitly discuss the direction and magnitude of potential biases?	6	x	

UOB Open

15.	Were the conclusions/recommendations of the study justified and based on the study results?	8	x	
16.	Was there a statement disclosing the source of funding for the study?	3	x	
Total score		100	89	

Quality of Health Economic Studies (QHES) instrument – Thomas et al 2021

	Questions	Points available	Yes	No
1.	Was the study objective presented in a clear, specific, and measurable manner?	7	x	
2.	Were the perspective of the analysis (societal, third-party payer, etc.) and reasons for its selection stated?	4	x	
3.	Were variable estimates used in the analysis from the best available source (RCT = best, expert opinion = worst)?	8	x	
4.	If estimates came from a subgroup analysis, were the groups pre-specified at the beginning of the study?	1	x (not applicable)	
5.	Was uncertainty handled by (1) statistical analysis to address random events, (2) sensitivity analysis to cover a range of assumptions?	9	x	
6.	Was incremental analysis performed between alternatives for resources and costs?	6	x	
7.	Was the methodology for data extraction (including the value of health states and other benefits) stated?	5	x	
8.	Did the analytic horizon allow time for all relevant and important outcomes? Were benefits and costs that went beyond 1 year discounted (3% - 5%) and justification given for the discount rate?	7	x	
9.	Was the measurement of costs appropriate and the methodology for the estimation of quantities and unit costs clearly described?	8	x	
10.	Was the primary outcomes measure(s) for the economic evaluation clearly stated and were the major short-term, long-term and negative outcomes included?	6		x
11.	Were the health outcomes measures/scales valid and reliable? If previously tested valid and reliable measures were not available, was justification given for the measures/scales used?	7	x	
12.	Were the economic model (including structure), study methods and analysis, and the components of the numerator and denominator displayed in a clear, transparent manner?	8	x	
13.	Were the choice of economic model, main assumptions, and limitations of the study stated and justified?	7	x	
14.	Did the author(s) explicitly discuss the direction and magnitude of potential biases?	6	x	
15.	Were the conclusions/recommendations of the study justified and based on the study results?	8	x	
16.	Was there a statement disclosing the source of funding for the study?	3	x	
Total score		100	94	

Quality of Health Economic Studies (QHES) instrument – Wong et al 2021

	Questions	Points available	Yes	No
1.	Was the study objective presented in a clear, specific, and measurable manner?	7	x	
2.	Were the perspective of the analysis (societal, third-party payer, etc.) and reasons for its selection stated?	4	x	
3.	Were variable estimates used in the analysis from the best available source (RCT = best, expert opinion = worst)?	8	x	
4.	If estimates came from a subgroup analysis, were the groups pre-specified at the beginning of the study?	1	x (not applicable)	
5.	Was uncertainty handled by (1) statistical analysis to address random events, (2) sensitivity analysis to cover a range of assumptions?	9	x	
6.	Was incremental analysis performed between alternatives for resources and costs?	6	x	
7.	Was the methodology for data extraction (including the value of health states and other benefits) stated?	5		X (choice of perfect health for the healthy state not justified in paper)
8.	Did the analytic horizon allow time for all relevant and important outcomes? Were benefits and costs that went beyond 1 year discounted (3% - 5%) and justification given for the discount rate?	7	x	
9.	Was the measurement of costs appropriate and the methodology for the estimation of quantities and unit costs clearly described?	8		x (costs are from an unpublished PhD thesis by lead author and are not otherwise described)
10.	Was the primary outcomes measure(s) for the economic evaluation clearly stated and were the major short-term, long-term and negative outcomes included?	6		x
11.	Were the health outcomes measures/scales valid and reliable? If previously tested valid and reliable measures were not available, was justification given for the measures/scales used?	7	x	
12.	Were the economic model (including structure), study methods and analysis, and the components of the numerator and denominator displayed in a clear, transparent manner?	8		X (no remission or transition between states modelled)

UOB Open

13.	Were the choice of economic model, main assumptions, and limitations of the study stated and justified?	7		X (no justification for not modelling remission or progression between cancer stages)
14.	Did the author(s) explicitly discuss the direction and magnitude of potential biases?	6	x	
15.	Were the conclusions/recommendations of the study justified and based on the study results?	8	x	
16.	Was there a statement disclosing the source of funding for the study?	3	x	
Total score		100	66	

Quality of Health Economic Studies (QHES) instrument – Callender et al 2021

	Questions	Points available	Yes	No
1.	Was the study objective presented in a clear, specific, and measurable manner?	7	x	
2.	Were the perspective of the analysis (societal, third-party payer, etc.) and reasons for its selection stated?	4	x	
3.	Were variable estimates used in the analysis from the best available source (RCT = best, expert opinion = worst)?	8	x	
4.	If estimates came from a subgroup analysis, were the groups pre-specified at the beginning of the study?	1	x (not applicable)	
5.	Was uncertainty handled by (1) statistical analysis to address random events, (2) sensitivity analysis to cover a range of assumptions?	9	x	
6.	Was incremental analysis performed between alternatives for resources and costs?	6	x	
7.	Was the methodology for data extraction (including the value of health states and other benefits) stated?	5	x	
8.	Did the analytic horizon allow time for all relevant and important outcomes? Were benefits and costs that went beyond 1 year discounted (3% - 5%) and justification given for the discount rate?	7	x	
9.	Was the measurement of costs appropriate and the methodology for the estimation of quantities and unit costs clearly described?	8	x	
10.	Was the primary outcomes measure(s) for the economic evaluation clearly stated and were the major short-term, long-term and negative outcomes included?	6		x
11.	Were the health outcomes measures/scales valid and reliable? If previously tested valid and reliable measures were not available, was justification given for the measures/scales used?	7	x	
12.	Were the economic model (including structure), study methods and analysis, and the components of the numerator and denominator displayed in a clear, transparent manner?	8	x	
13.	Were the choice of economic model, main assumptions, and limitations of the study stated and justified?	7	x	
14.	Did the author(s) explicitly discuss the direction and magnitude of potential biases?	6	x	
15.	Were the conclusions/recommendations of the study justified and based on the study results?	8	x	
16.	Was there a statement disclosing the source of funding for the study?	3		x (discloses conflicts but not explicitly specific funding)

UOB Open

Total score	100	91	

Quality of Health Economic Studies (QHES) instrument – Cenin et al, 2020

	Questions	Points available	Yes	No
1.	Was the study objective presented in a clear, specific, and measurable manner?	7	x	
2.	Were the perspective of the analysis (societal, third-party payer, etc.) and reasons for its selection stated?	4	x	
3.	Were variable estimates used in the analysis from the best available source (RCT = best, expert opinion = worst)?	8	x	
4.	If estimates came from a subgroup analysis, were the groups pre-specified at the beginning of the study?	1	x (not applicable)	
5.	Was uncertainty handled by (1) statistical analysis to address random events, (2) sensitivity analysis to cover a range of assumptions?	9	x	
6.	Was incremental analysis performed between alternatives for resources and costs?	6	x	
7.	Was the methodology for data extraction (including the value of health states and other benefits) stated?	5		x (Baseline utility data and sources not reported)
8.	Did the analytic horizon allow time for all relevant and important outcomes? Were benefits and costs that went beyond 1 year discounted (3% - 5%) and justification given for the discount rate?	7	x	
9.	Was the measurement of costs appropriate and the methodology for the estimation of quantities and unit costs clearly described?	8	x	
10.	Was the primary outcomes measure(s) for the economic evaluation clearly stated and were the major short-term, long-term and negative outcomes included?	6		x
11.	Were the health outcomes measures/scales valid and reliable? If previously tested valid and reliable measures were not available, was justification given for the measures/scales used?	7	x	
12.	Were the economic model (including structure), study methods and analysis, and the components of the numerator and denominator displayed in a clear, transparent manner?	8	x	
13.	Were the choice of economic model, main assumptions, and limitations of the study stated and justified?	7	x	
14.	Did the author(s) explicitly discuss the direction and magnitude of potential biases?	6	x	
15.	Were the conclusions/recommendations of the study justified and based on the study results?	8	x	
16.	Was there a statement disclosing the source of funding for the study?	3	x	

UOB Open

Total score	100	89	

Quality of Health Economic Studies (QHES) instrument – Naber et al, 2019

	Questions	Points available	Yes	No
1.	Was the study objective presented in a clear, specific, and measurable manner?	7	x	
2.	Were the perspective of the analysis (societal, third-party payer, etc.) and reasons for its selection stated?	4	x	
3.	Were variable estimates used in the analysis from the best available source (RCT = best, expert opinion = worst)?	8	x	
4.	If estimates came from a subgroup analysis, were the groups pre-specified at the beginning of the study?	1	x	
5.	Was uncertainty handled by (1) statistical analysis to address random events, (2) sensitivity analysis to cover a range of assumptions?	9	x	
6.	Was incremental analysis performed between alternatives for resources and costs?	6	x	
7.	Was the methodology for data extraction (including the value of health states and other benefits) stated?	5		x (Baseline utility data and sources not reported)
8.	Did the analytic horizon allow time for all relevant and important outcomes? Were benefits and costs that went beyond 1 year discounted (3% - 5%) and justification given for the discount rate?	7	x	
9.	Was the measurement of costs appropriate and the methodology for the estimation of quantities and unit costs clearly described?	8	x	
10.	Was the primary outcomes measure(s) for the economic evaluation clearly stated and were the major short-term, long-term and negative outcomes included?	6		x
11.	Were the health outcomes measures/scales valid and reliable? If previously tested valid and reliable measures were not available, was justification given for the measures/scales used?	7	x	
12.	Were the economic model (including structure), study methods and analysis, and the components of the numerator and denominator displayed in a clear, transparent manner?	8	x	
13.	Were the choice of economic model, main assumptions, and limitations of the study stated and justified?	7	x	
14.	Did the author(s) explicitly discuss the direction and magnitude of potential biases?	6	x	
15.	Were the conclusions/recommendations of the study justified and based on the study results?	8	x	
16.	Was there a statement disclosing the source of funding for the study?	3	x	
Total score		100	89	

Quality of Health Economic Studies (QHES) instrument – Callender et al 2019

	Questions	Points available	Yes	No
1.	Was the study objective presented in a clear, specific, and measurable manner?	7	x	
2.	Were the perspective of the analysis (societal, third-party payer, etc.) and reasons for its selection stated?	4	x	
3.	Were variable estimates used in the analysis from the best available source (RCT = best, expert opinion = worst)?	8	x	
4.	If estimates came from a subgroup analysis, were the groups pre-specified at the beginning of the study?	1	x	
5.	Was uncertainty handled by (1) statistical analysis to address random events, (2) sensitivity analysis to cover a range of assumptions?	9	x	
6.	Was incremental analysis performed between alternatives for resources and costs?	6		x (based on O'Mahony https://hrbopenresearch.org/articles/3-23)
7.	Was the methodology for data extraction (including the value of health states and other benefits) stated?	5	x	
8.	Did the analytic horizon allow time for all relevant and important outcomes? Were benefits and costs that went beyond 1 year discounted (3% - 5%) and justification given for the discount rate?	7	x	
9.	Was the measurement of costs appropriate and the methodology for the estimation of quantities and unit costs clearly described?	8	x	
10.	Was the primary outcomes measure(s) for the economic evaluation clearly stated and were the major short-term, long-term and negative outcomes included?	6		x
11.	Were the health outcomes measures/scales valid and reliable? If previously tested valid and reliable measures were not available, was justification given for the measures/scales used?	7	x	
12.	Were the economic model (including structure), study methods and analysis, and the components of the numerator and denominator displayed in a clear, transparent manner?	8	x	
13.	Were the choice of economic model, main assumptions, and limitations of the study stated and justified?	7	x	

UOB Open

14.	Did the author(s) explicitly discuss the direction and magnitude of potential biases?	6	x	
15.	Were the conclusions/recommendations of the study justified and based on the study results?	8	x	
16.	Was there a statement disclosing the source of funding for the study?	3	x	
Total score		100	88	

Quality of Health Economic Studies (QHES) instrument – Pashayan et al, 2018

	Questions	Points available	Yes	No
1.	Was the study objective presented in a clear, specific, and measurable manner?	7	x	
2.	Were the perspective of the analysis (societal, third-party payer, etc.) and reasons for its selection stated?	4	x	
3.	Were variable estimates used in the analysis from the best available source (RCT = best, expert opinion = worst)?	8	x	
4.	If estimates came from a subgroup analysis, were the groups pre-specified at the beginning of the study?	1	x	
5.	Was uncertainty handled by (1) statistical analysis to address random events, (2) sensitivity analysis to cover a range of assumptions?	9	x	
6.	Was incremental analysis performed between alternatives for resources and costs?	6	x	
7.	Was the methodology for data extraction (including the value of health states and other benefits) stated?	5	x	
8.	Did the analytic horizon allow time for all relevant and important outcomes? Were benefits and costs that went beyond 1 year discounted (3% - 5%) and justification given for the discount rate?	7	x	
9.	Was the measurement of costs appropriate and the methodology for the estimation of quantities and unit costs clearly described?	8	x	
10.	Was the primary outcomes measure(s) for the economic evaluation clearly stated and were the major short-term, long-term and negative outcomes included?	6		x
11.	Were the health outcomes measures/scales valid and reliable? If previously tested valid and reliable measures were not available, was justification given for the measures/scales used?	7	x	
12.	Were the economic model (including structure), study methods and analysis, and the components of the numerator and denominator displayed in a clear, transparent manner?	8	x	
13.	Were the choice of economic model, main assumptions, and limitations of the study stated and justified?	7	x	
14.	Did the author(s) explicitly discuss the direction and magnitude of potential biases?	6	x	
15.	Were the conclusions/recommendations of the study justified and based on the study results?	8	x	

16.	Was there a statement disclosing the source of funding for the study?	3	x	
Total score		100	94	
